# Effects of Dietary Rumen-Protected Glucose and Rumen-Protected Taurine Levels on Growth Performance, Serum Biochemical Indicators, and Liver Health in Yaks

**DOI:** 10.3390/ani15081152

**Published:** 2025-04-17

**Authors:** Yuanyuan Chen, Xiaolin Wang, Lianghao Lu, Bao Zhang, Huaming Yang, Shoupei Zhao, Zhisheng Wang, Lizhi Wang, Quanhui Peng, Bai Xue

**Affiliations:** Animal Nutrition Institute, Sichuan Agricultural University, Chengdu 611130, China; cyy2022214041@stu.sicau.edu.cn (Y.C.); wxl547312630@163.com (X.W.); 13551012639@163.com (L.L.); 2022314074@stu.sicau.edu.cn (B.Z.); 13980277829@163.com (H.Y.); 2023114011@stu.sicau.edu.cn (S.Z.); zswangsicau@126.com (Z.W.); 1285@sicau.edu.cn (L.W.); pengquanhui@126.com (Q.P.)

**Keywords:** liver antioxidant, rumen-protected glucose, rumen-protected taurine, yak

## Abstract

Yaks are an important livestock species on the Tibetan Plateau, but traditional grazing practices cause a sharp drop in their weight during winter, leading to grassland degradation due to overgrazing. Although off-site fattening can improve performance and protect ecology, it often leads to a negative energy balance, liver metabolism disorders, and immune impairment due to stress. However, the effects of rumen-protected glucose (RPG) and rumen-protected taurine (RPT) on yak liver health are not yet clear. The purpose of this study was to evaluate the effects of dietary RPG and RPT levels on the growth performance, serum biochemical parameters, liver antioxidant capacity, and immunity of yaks. Twenty-eight healthy yaks weighing 192.7 ± 20.6 kg were randomly divided into four treatments: LGLT (RPG: 1%—low RPG [LG]; RPT: 5 g/d—low RPT [LT]), LGHT (RPG: 1%—low RPG [LG]; RPT: 20 g/d—high RPT [HT]), HGLT (RPG: 3%—high RPG [HG]; RPT: 5 g/d—low RPT [LT]), and HGHT (RPG: 3%—high RPG [HG]; RPT: 20 g/d—high RPT [HT]). The results showed that compared with the LTHT treatment group, the HGHT group upregulated the serum concentrations of glucose (*p* = 0.004) and Interleukin-10 (*p* = 0.03), the relative mRNA expression of small heterodimer partners (*p* = 0.01), and the sterol 12-alpha-hydroxylase (*p* < 0.001), while reducing the serum concentration of gamma-glutamyl transferase (*p* = 0.048). The serum concentration of hepatic protein carbonyl (*p* = 0.005) and malondialdehyde (*p* = 0.03) was lower in the LGHT and HGHT treatment groups than in the LGLT and HGLT groups. The relative mRNA expression of Toll-like receptor 4 (*p* = 0.02), Interleukin-8 (*p* < 0.01), and Interleukin-1β (*p* < 0.01) was lower in the LGHT and HGHT groups than in the LGLT and HGLT groups. Tumor necrosis factor expression was lower (*p* = 0.04) and glucose transporter 2 expression was higher (*p* < 0.01) in the HGHT group compared to other treatment groups. The expression of glucokinase, glycogen synthase, pyruvate kinase, and farnesoid X receptor was higher in the HGLT treatment group than in other treatments (*p* < 0.01). In conclusion, dietary supplementation with 3% PRG and 5 g/d PRT can enhance liver antioxidant capacity and immune function, reduce lipid peroxidation, and promote glucose and bile acid metabolism in yaks.

## Simple Summary

Yaks typically inhabit the Tibetan Plateau and its surrounding areas at altitudes above 3000 m (sub-alpine regions), where they may face harsh conditions or prolonged periods of starvation. Therefore, yak farming is facing various challenges, which can easily lead to stress injuries in the production process. Rumen-protected glucose (RPG) can increase the energy density of the diet without affecting rumen fermentation and feed intake, as well as rapidly improving energy supply. Rumen-protected taurine (RPT) can improve animal performance and immunity, as well as relieving oxidative stress. The aim of this study was to investigate the effects of RPG and RPT levels on the growth performance, serum biochemical indices, and liver health of yaks. The results showed that dietary supplementation of RPG and RPT could promote liver metabolism, improving the immunity capacity of yaks. The results of this study are important references for the application of RPG and RPT in yak farming.

## 1. Introduction

The yak (*Bos grunniens*) is a unique species of livestock that is endemic to high-altitude regions, particularly the Tibetan Plateau in China [1]. Yaks provide a range of benefits to local communities, including meat, milk, and transportation, thus playing a vital role in the local economy and livestock husbandry [2]. During the prolonged cold season, grazing yaks experience significant weight loss (even exceeding a lost of 25% of their body weight) due to the scarcity of high-quality forage [3]. Moreover, the increase in the yak population has led to overgrazing, which has exacerbated grassland degradation and put pressure on the ecological environment. Therefore, the off-site fattening model for yaks can enhance their production performance and promote ecological protection. However, significant changes in climate and altitude can lead to stress responses, resulting in a negative energy balance, disordered hepatic glycogen and lipid metabolism, and impaired immune function [4].

Rumen-protected additives are composite products that are coated with special materials such as fatty acids and novel composite materials to prevent fermentation in the rumen; these can be rapidly and efficiently absorbed and utilized in the hindgut. Rumen-protected glucose (RPG) is a relatively safe energy source for yaks, providing an effective glucose supply while maintaining rumen stability. RPG can be released directly from the small intestine into circulation because it is covered with special substances such as fatty acids that prevent its fermentation and degradation in the rumen [5]. RPG can effectively alleviate the negative energy balance in animals during early lactation [6]. In addition, the supplementation of RPG can significantly increase milk production in dairy cows, while also alleviating inflammatory responses [7]. By blocking lipolysis and gluconeogenesis, RPG helps improve the energy absorption and metabolic efficiency of animals, thereby effectively regulating carbohydrate and lipid metabolism, as well as reducing the occurrence of metabolic diseases [8].

Taurine is one of the most abundant free amino acids in the human body and has a wide range of biological roles, including participation in glycolipid metabolism, anti-inflammatory and antioxidant properties, and membrane stabilization [9]. Taurine supplementation enhances antioxidant capacity, alleviates lipopolysaccharide (LPS)-induced inflammatory response and oxidative stress [10], reduces inflammatory reactions, and decreases levels of inflammatory markers in animals [11]. Taurine can also alleviate tissue damage caused by hyperglycemia and improve blood glucose stability [12]. The addition of taurine to high-fat diets improves antioxidant capacity and immunity in *Monopterus albus* [13]. In addition, our previous research found that rumen-protected taurine (RPT) can enhance the antioxidant capacity of yaks during transportation, thereby alleviating inflammatory damage [5]. Taurine may enhance animal growth and health through these mechanisms, though its mechanisms of action in yak liver remain to be fully elucidated.

At present, the effects of RPG and RPT on yak liver health remain unclear. Our hypothesis is that the synergistic effect of RPG and RPT enhances the antioxidant capacity of yaks and promotes liver health. Our study aimed to investigate the effects of dietary RPG and RPT on the growth performance, serum biochemical indices, and liver antioxidant capacity of yaks.

## 2. Materials and Methods

### 2.1. Animals and Experimental Design

All animals in this study were treated in compliance with the Chinese Animal Welfare Guidelines, and the experimental protocol was approved by the Experimental Animal Committee of Animal Nutrition Institute, Sichuan Agricultural University (No. SCAUAC201936).

A total of 28 healthy 2.5-year-old male yaks, weighing 192.7 ± 20.6 kg, were randomly divided into 4 treatments, with 7 replicates in each group and 1 yak in each replicate. They were divided into four treatment groups with different levels of RPG (1% and 3%) and RPT (5 and 20 g/d) as follows: LGLT (RPG: 1%—low RPG [LG]; RPT: 5 g/d—low RPT [LT]), LGHT (RPG: 1%—low RPG [LG]; RPT: 20 g/d—high RPT [HT]), HGLT (RPG: 3%—high RPG [HG]; RPT: 5 g/d—low RPT [LT]), and HGHT (RPG: 3%—high RPG [HG]; RPT: 20 g/d—high RPT [HT]). Before the commencement of the experiment, the test site was thoroughly disinfected and the test animals were individually numbered and dewormed. Yaks were housed individually in pens at the Animal Nutrition Institute’s Research Farm, Sichuan Agricultural University (Yaan, China). The daily total mixed ration (TMR), along with RPG and RPT, was divided into two equal portions and was administered twice daily at 09:00 and 18:00, with free access to water provided throughout the experimental period. To ensure the complete intake of the additives, a phased feeding protocol was implemented, whereby the TMR-containing additives were provided first, before the remaining basal TMR was supplied after the full consumption of the additive-containing ration. The test personnel were familiar with the feeding and management process of the test yaks; according to the feed intake of the previous day, the feeding amount of the next day was adjusted to ensure that there was about 10 % of the remaining material in the feeding tank. Following the 14-day adaptation period, a 63-day trial experiment was conducted. The composition and nutritional levels of yak diets are shown in Table 1. The diet was formulated according to the NRC (2007) [14] to meet the nutritional requirements of yaks. The RPG (provided by Shanghai Menon Biological Technology Co., Ltd., Shanghai, China) and RPT (provided by Qingdao Kailide Biological Technology Co., Ltd., Qingdao, China) used in this study both contained 50% glucose and 50% taurine, with the products demonstrating a small intestine release rate of 90 %. The actual rumen bypass rates of RPG and RPT were determined as 88% and 95.7%, respectively, through in vitro rumen fermentation conducted using the first step of the two-stage digestion method by Tilley and Terry [15].

### 2.2. Determination of Growth Performance

Yaks were weighed and recorded on days 0 and 63 of the experiment. This ensured that the amount of leftover feed on the same day was 10%; then, the feed intake of the test yaks in each group was recorded, and the average daily gain (ADG), dry matter intake (DMI), and feed-to-weight ratio (F/G) were calculated.

### 2.3. Sample Collection

On the 62nd day of the experiment, jugular venous blood samples were collected from all the experimental yaks using vacuum blood collection tubes 3 h before morning feeding. The collected blood samples were placed in a centrifuge and were centrifuged at 3000× *g* for 15 min at 4 °C. The serum was stored at −20 °C for the subsequent analysis of serum biochemical and immune-related indicators.

### 2.4. Collection and Determination of Organ Samples

After the trial period, 7 yaks from each treatment group were slaughtered, totaling 28 yaks. After 16 h of fasting, the jugular vein was bled to death, and the heart, liver, spleen, and kidneys were collected and dried with absorbent paper and weighed separately. Furthermore, part of the liver tissue was snipped with surgical scissors, rinsed gently with PBS, and placed in a 2 mL frozen storage tube, before being stored at −80 °C for liver antioxidant and related genetic determination. The organ index was calculated using the following formula: Organ index (%) = 100% × organ weight/living weight.

### 2.5. Determination of Serum Biochemical and Immune Indexes

Serum concentrations of glucose (GLU), total bile acid (TBA), alkaline phosphatase (ALP), glutamyl transpeptidase (GGT), total protein (TP), albumin (ALB), aspartate aminotransferase (AST), total cholesterol (TC), lactate dehydrogenase (LDH), high-density lipoprotein (HDCL), and low-density lipoprotein (LDLC) were measured using an automatic biochemical analyzer (Hitachi 3100, Tokyo, Japan). The serum immune indexes, as well as the levels of Tumor necrosis factor (TNF-α), Interleukin-6 (IL-6), Interleukin-1β (IL-1β), Interleukin-4 (IL-4), and Interleukin-10 (IL-10), were measured using an Elisa kit. The kit was purchased from Jiangsu Enzyme Immunobiological Reagent Co., Ltd. (Nanjing, China) and was used in accordance with the manufacturer’s protocol.

### 2.6. Determination of Liver Antioxidant Index

The activities of glutathione peroxidase (GSH-Px), total superoxide dismutase (T-SOD), and catalase (CAT), as well as total antioxidant capacity (T-AOC), alkaline phosphatase (ALP), malonaldehyde (MDA), and protein carbonyl (PCO) concentrations, were measured with GSH-Px kits (A005-1), T-SOD kits (A001-1), a CAT kit (A007-1-1), a T-AOC kit (A015-3-1), an MDA kit (A003-1), and a PCO kit (A087-1). All the test kits were purchased from Nanjing Jiancheng (Nanjing, China). The measurements of aspartate aminotransferase (AST) and alanine aminotransferase (ALT) activities were conducted with an AST assay kit (G0424W) and an ALT assay kit (G0423W), which were provided by Geruisi-bio (Suzhou, China). Operations were conducted following the manufacturer’s instructions.

### 2.7. Quantitative Real-Time PCR Analysis

Total RNA from yak liver was extracted with TRIzol reagent. RNA concentration and purity were measured using the DNA/RNA Calculator. The liver RNA was then reverse-transcribed into cDNA (ExonScript^®^ RT SuperMix with dsDNase, Rong Gene, Chengdu, China). The NCBI website was used to design the primer sequence, which was synthesized by Shenggong Bioengineering (Shanghai, China) Co., Ltd. The primer sequences and amplification efficiencies for real-time quantitative PCR (qPCR) are shown in Table 2. SYBR (2×Universal Blue SYBR Green qPCR Master Mix) was used as the dye. The expression of liver-related genes was measured using QuantStudioTM5 quantitative PCR (Thermo Fisher Scientific, Singpore). The β-actin gene was employed as the internal reference gene, and the relative expression of the target gene was calculated using the 2^−∆∆CT^ method.

### 2.8. Statistical Analysis

Normality and variance chi-square tests were performed on all data before comparing treatments. SPSS 27.0 software was used to analyze the effects of RPG and RPT addition levels on the growth performance, blood biochemistry, and liver antioxidant indexes of yaks using two-way ANOVA. When significant interactions between dietary RPG and RPT levels were observed, one-way ANOVA combined with Duncan’s multiple comparison test was performed. All data are expressed as mean ± SEM. *p* < 0.05 indicated a significant difference; *p* < 0.01 indicated an extremely significant difference; and *p* > 0.05 indicated no significant difference.

## 3. Results

### 3.1. Growth Performance

The level of RPG and RPT in the diet did not affect the final BW, ADG, DMI, and F/G in yaks (*p* > 0.05; Table 3). In addition, there was no significant interaction between RPG and RPT regarding the final weight, ADG, DMI, and F/G (*p* > 0.05).

### 3.2. Organ Index

Table 4 shows that the addition of RPG and RPT to the diets had no significant effect on liver, heart, kidneys, and spleen weight, as well as liver index, heart index, kidney index, and spleen index in yaks (*p* > 0.05).

### 3.3. Serum Biochemical Indexes

The effects of dietary RPG and RPT on the serum biochemical indices of yaks are shown in Table 5. Increasing the dietary RPG level increased the serum GLU concentration (*p* = 0.004) but decreased GGT concentration (*p* = 0.048). The serum LDH concentration was lower in the HGHT treatment group than in the LGHT group (*p* = 0.034). Dietary levels of RPG and RPT did not affect other biochemical markers in serum (*p* > 0.05).

### 3.4. Serum Immune Indices

Increasing the dietary RPG level decreased the serum IL-10 concentration (*p* = 0.032; Table 6). However, there was no significant difference in IL-1β, TNF-α, IL-6, and IL-4 when increasing the levels of RPG and RPT (*p* > 0.05).

### 3.5. Hepatic Antioxidation Capacity

The effects of RPG and RPT on hepatic antioxidant capacity are shown in Table 7. Increasing the level of RPT reduced the concentration of PCO (*p* = 0.005) and MDA (*p* = 0.027). There were no significant differences in hepatic AST, ALT, GSH-Px, CAT, SOD, and T-AOC activities (*p* > 0.05).

### 3.6. Hepatic Inflammatory

**Figure 1:**
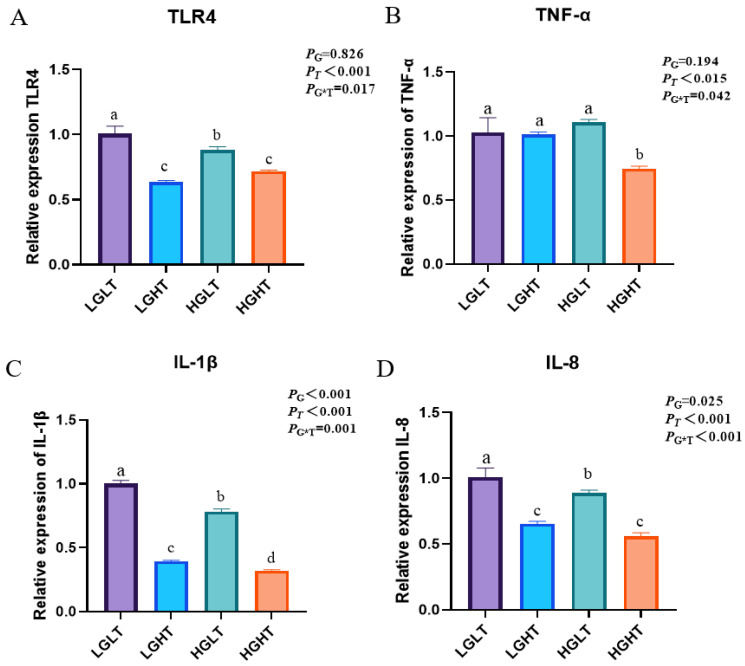
Effects of RPG and RPT levels on inflammatory factors in yak live (*n* = 6). “G” represents RPG; “T” represents RPT; and “G*T” represents the interaction effect of RPG and RPT. (**A**) The relative expression of TLR4 in the liver. (**B**) The relative expression of TNF-α in the liver. (**C**) The relative expression of IL-1β in the liver. (**D**) The relative expression of IL-8 in the liver. Note: Values are mean ± SEM; different letters indicate significant differences (*p* < 0.05). Different colors represent different treatment groups.

The effects of RPG and RPT on the levels of inflammatory factors in yak liver are shown in Figure 1. The relative mRNA expression of TLR4 and IL-8 was lower in the LGHT and HGHT treatment groups than in the LGLT and HGLT groups (*p* < 0.05). The TNF-α expression was lower in the HGHT treatment group than in the other groups (*p* = 0.042). The IL-1β expression was lower in the LGHT and HGHT groups than in the LGLT and HGLT groups (*p* = 0.001).

### 3.7. Hepatic Glucose Metabolism

**Figure 2:**
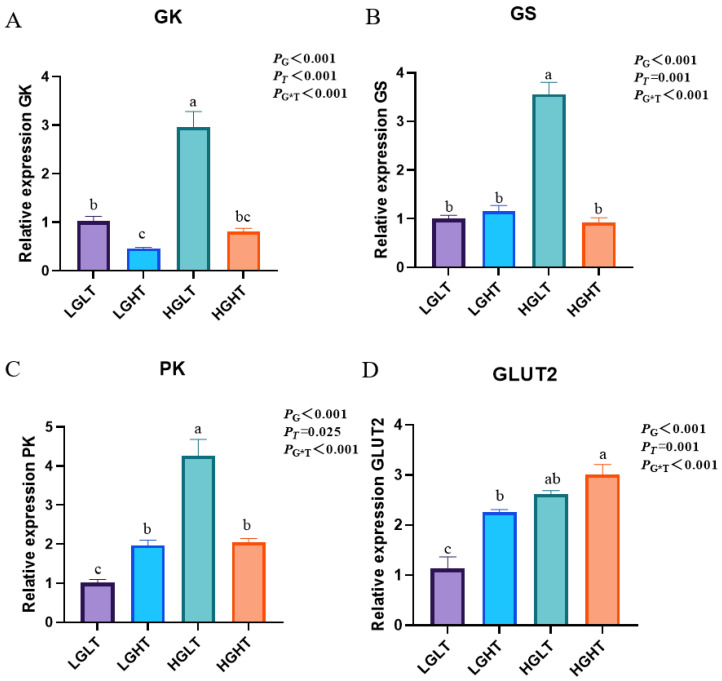
Effects of RPG and RPT levels on hepatic glucose metabolism in yaks (*n* = 6). “G” represents RPG; “T” represents RPT; and “G*T” represents the interaction effect of RPG and RPT. (**A**) The relative expression of GK in the liver. (**B**) The relative expression of GS in the liver. (**C**) The relative expression of PK in the liver. (**D**) The relative expression of GLUT2 in the liver. Note: Values are mean ± SEM; different letters indicate significant differences (*p* < 0.05). Different colors represent different treatment groups.

Figure 2 demonstrates the levels of hepatic glucose metabolism genes in yaks. The GK, GS, and PK expressions were lower in other treatment groups than in the HGLT group (*p* < 0.001). The GLUT2 expression was lower in other treatment groups than in the HGHT group (*p* < 0.001).

### 3.8. Hepatic Bile Acid Metabolism

**Figure 3:**
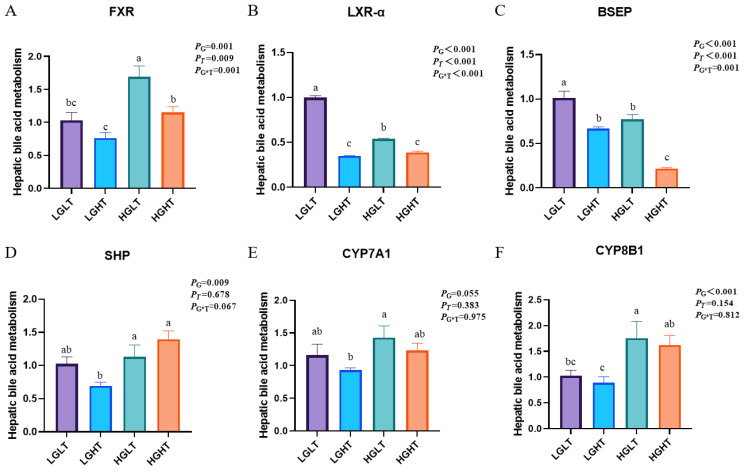
Effects of RPG and RPT levels on hepatic bile acid metabolism in yaks (*n* = 6). “G” represents RPG; “T” represents RPT; and “G*T” represents the interaction effect of RPG and RPT. (**A**) The relative expression of FXR in the liver. (**B**) The relative expression of LXR-α in the liver. (**C**) The relative expression of BSEP in the liver. (**D**) The relative expression of SHP in the liver. (**E**) The relative expression of CYP7A1 in the liver. (**F**) The relative expression of CYP8B1 in the liver. Note: Values are mean ± SEM; different letters indicate significant differences (*p* < 0.05). Different colors represent different treatment groups.

The expression levels of genes related to bile acid metabolism in the liver of yaks from different groups are shown in Figure 3. The relative mRNA expression of LXR-α and BSEP was lower in other treatment groups than in the LGLT group (*p* = 0.001). The expression of FXR was lower in other treatment groups than in the HGLT group (*p* = 0.001). We found that increasing RPG levels increased the expression of SHP and CYP8B1 (*p* < 0.001).

## 4. Discussion

In the process of animal production, growth performance is an important index. Zhang et al. [16] demonstrated that supplementing the diet with RPT can enhance the ADG of steers. Early glucose injection increased the body weight of *Oreochromis niloticus* at 12–16 weeks, thus improving the growth performance of the fish [17]. Dietary supplementation with 0.3% or 0.5% taurine improves the growth performance of weaned piglets by increasing the daily weight gain and the feed-to-gain ratio [18]. However, Wang et al. [5] found that supplementing yaks with RPG and RPT after long-distance transportation did not result in any changes in growth performance. In our study, increasing the concentrations of RPG and RPT in diets did not change the ADG, DMI, and F/G of yaks, suggesting that the dosage of RPG and RPT is not the main reason for affecting growth performance. We did not provide data on non-RPG and -RPT diets, so we cannot prove whether feeding these two additives improves yak growth performance compared with not feeding, which will be refined in future work.

The weight ratio of internal organs can be used as an index to measure the growth and development of animals and to determine whether there is disease [19]. The organ index is one of the most important indices to judge the health and development of animals, reflecting the total nutritional status of animals and the physiological function of internal organs. The data showed that there was no significant effect of organ weight and organ index of yak heart, liver, spleen, and kidney among different treatment groups, which indicates that the supplementation of diets with different levels of RPG and RPT had no adverse effect on the development of internal organs in yaks.

Serum biochemical indexes and immune factor levels can reflect the comprehensive metabolic capacity and overall immunity of animals to a certain extent. Enzymes and metabolites like ALP, AST, and GGT in serum are important indices to evaluate liver function [20,21,22]. In our study, we found that the supplementation of RPG significantly reduced the concentration of GGT, thus having a protective effect on yak liver. Hagar et al. [23] have showed that feeding taurine reduced the levels of serum GGT, suggesting that taurine protects against liver injury in rats by lowering serum GGT levels; there was no significant difference in the concentration of ALP and AST between different levels of RPG and RPT added to the diets, suggesting that different doses of RPG and RPT may not cause liver damage. Serum GLU is one of the principal energy substrates in animals [24]. In our study, dietary RPG supplementation increased serum GLU concentrations. Wang et al. reported that dietary supplementation with RPG elevated serum GLU concentrations in early-lactation cows, which is consistent with the present study, suggesting that RPG can rapidly provide efficient energy that is easily available to the organism [6,7]. In summary, serum GLU levels may be elevated, which enhances carbohydrate digestion and absorption in yaks following RPG supplementation [25]. This rapid supply of energy is crucial for the growth and maintenance of yaks in cold environments, particularly during the lactation period or growth stages when energy demands are high.

The liver’s antioxidant capacity is crucial for defending against oxidative stress and inflammation, which are key factors in the development of various liver diseases [26]. MDA reflects the degree of lipid oxidative damage between tissues [27]. In different oxidative modifications of proteins, the introduction of carbonyl groups such as aldehyde, ketone, and lactam into the amino acid side chains of proteins is a major hallmark for oxidative damage to proteins, and is termed “protein carbonylation” [28]. Rosemberg et al. [29] also found that 150 mg/L and 400 mg/L taurine could effectively inhibit lipid peroxidation when exploring the protective effect of taurine on zebrafish brain oxidative stress induced by ethanol. In addition, the supplementation of 2% taurine in the diet reduced the hepatic MDA content of juvenile turbot (*Scophthalmus maximus* L.) [30]. Similarly, in our study, increasing RPT concentration significantly reduced the contents of MDA and PCO, indicating that RPT promoted the scavenging ability of oxygen free radicals in the liver, reduced the production of lipid peroxides, and inhibited protein damage, thereby promoting yak liver health.

Inflammatory response is an important part of the innate immune system to respond to various stimuli; it is mainly mediated by cytokines. IL-10 is a multifunctional anti-inflammatory factor that mainly mediates inflammation and induces humoral immune response, thereby maintaining the normal function of the body [31]. Cantuária’s study showed that high glucose concentrations increased IL-10 production [32]. In this study, increasing dietary RPG concentration significantly increased IL-10 levels in serum, suggesting that RPG may improve anti-inflammatory capacity in yaks. TLR4 is a kind of pattern recognition receptor (PRR) on the cell surface, which plays an important role in the process of innate immunity [33]. IL-1β and TNF-α can induce inflammatory responses by regulating the expression of other cytokines [34]. Taurine has been shown to have anti-inflammatory effects in mammals [12,35]. Lin et al. reported that taurine could attenuate the inflammatory response by decreasing serum and hepatic IL-1β, IL-6, and TNF-α gene expression levels, as well as activating the TLR4/MyD88 signaling pathway in rats with chronic alcohol consumption [36]. Ma et al. found that taurine improved lung injury by decreasing neutrophil infiltration and IL-1β production [37]. The results of this study showed that increasing the dietary RPT concentration significantly reduced the expression levels of hepatic TLR4 and IL-1β genes. This suggests that increasing dietary RPT concentration significantly improves immunity in yaks. IL-8 is a novel leukocyte chemotactic cytokine produced by a variety of cells in response to inflammatory stimuli, which acts as a chemical signal to attract neutrophils to sites of inflammation [38]. In this study, increasing dietary RPG and RPT levels significantly reduced IL-8 gene expression. This study demonstrates that dietary supplementation with RPT and RPG can significantly reduce the expression of pro-inflammatory cytokines and improve immune function in yaks, thereby enhancing liver health and overall resistance to disease.

Sugar metabolism plays a significant role in biological metabolism [39]. In particular, glycolysis, as a multifaceted metabolic pathway and signaling hub, plays a crucial role in energy supply, cell metabolism, growth regulation, and disease development [40]. In the liver, glucokinase (GK) increases the uptake of glucose by the liver and the synthesis of glycogen [41]. Glycogen synthase (GS) is the rate-limiting enzyme for hepatic glycogen synthesis [42]. Pyruvate kinase (PK) is a glycolytic enzyme that catalyzes the final step of glycolysis [43]. Zhang et al. [44] showed that high dietary starch levels inhibit glycolysis and hinder glucose utilization in largemouth bass *Micropterus salmoides*. In this experiment, the expression of GK, GS, and PK in the HGLT group was also significantly higher than that in other groups. This may be due to the activation of FXR increasing the expression of GK and PK genes [45]; additionally, elevated serum GLU levels increased GS expression in yak liver and promoted liver glycogen synthesis [46]. This suggests that increased levels of RPG promote hepatic glycolysis and glycogen synthesis in yaks on diets containing moderate amounts of RPT.

Bile acids are produced from cholesterol in hepatocytes and undergo enterohepatic circulation between the liver and intestine [47,48]. Bile acid homeostasis is tightly regulated through a multistep feedback loop of the farnesoid X receptor (FXR) and small heterodimer partner (SHP). High glucose concentrations increase FXR O-GlcN acylation [49]. FXR activation reduces the intracellular bile acid load in target tissues by inhibiting bile salt export pumps (e.g., BSEPs) [47]. In the present study, increasing the concentration of RPG significantly increased the expression of FXR and SHP, as well as decreasing the expression of BSEPs. This suggests that the RPG activation of FXR reduces liver bile acid load by inhibiting BSEPs. Based on the above results, increasing RPG levels in diets containing moderate amounts of RPT improves hepatic bile acid metabolism and promotes liver health in yaks.

## 5. Conclusions

In conclusion, increasing the levels of RPG and RPT in yak diets did not improve growth performance or organ index. However, dietary supplementation with 3% RPG and 5 g/d RPT enhanced liver antioxidant capacity and immune function, reduced lipid peroxidation, and promoted glucose and bile acid metabolism in yaks.

## Figures and Tables

**Table 1:** Composition and nutrient levels of experimental diet (DM basis).

Item	LG		HG	
	LT (5 g/d)	HT (20 g/d)	LT (5 g/d)	HT (20 g/d)
**Ingredients % DM**				
Corn	15.5	15.5	14.7	14.7
Wheat bran	5.6	5.6	5.0	5.0
Soybean meal	9.0	9.0	9.4	9.4
Rapeseed meal	3.0	3.0	3.0	3.0
Palm oil	2.0	2.0	1.0	1.0
Straw	35.0	35.0	35.0	35.0
Leymus chinensis	27.0	27.0	27.0	27.0
Calcium hydrogen phosphate	0.1	0.1	0.1	0.1
Calcium carbonate	0.6	0.6	0.6	0.6
Sodium chloride	0.2	0.2	0.2	0.2
Premix ^1^	1.0	1.0	1.0	1.0
Rumen-protected glucose	1.0	1.0	3.0	3.0
**Nutrient level ^2^ % DM**				
Neg, MJ/kg	5.97	5.97	5.97	5.97
CP	9.88	9.88	9.82	9.82
NDF	46.2	46.2	45.9	45.9
ADF	30.7	30.7	30.6	30.6
Ca	0.52	0.52	0.50	0.50
P	0.33	0.33	0.34	0.34

^1^ The premix provides the following per kg of the diet: Cu—10 mg; Fe—40 mg; Mn—60 mg; Zn—60 mg; Se—0.3 mg; I—0.5 mg; Co—0.1 mg; VA—4400 IU; VD—31300 IU; VE—20 IU. NEg is calculated, the rest are measured. ^2^ Abbreviations: NEg—net energy for grain; CP—crude protein; NDF—neutral detergent fiber; ADF—acid detergent fiber.

**Table 2:** Primer sequences.

Gene	Primer Sequences (5′–3′)	Accession Number	Amplification Efficiency (%)
*TLR4*	F: GGATGAAGACTGGGTGCGGAATG	XM_005891938.1	102
	R: CTGGATGATATTGGCGGCGATGG		
*IL-8*	F: GCTGGCTGTTGCTCTCTTGGC	XM_005891246.2	108
	R: GGGGTGGAAAGGTGTGGAATGTG		
*TNF-α*	F: CTGGCGGAGGAGGTGCTCTC	XM_005904178.1	105
	R: GGAGGAAGGAGAAGAGGCTGAGG		
*IL-1β*	F: ATGAAGAGCTGCATCCAACACCTG	NM_174093.1	101
	R: ACCGACACCACCTGCCTGAAG		
*GS*	F: CTGGCTGAGGGTGTGTTGCTG	XM_005900427.2	106
	R: CGGTGAAGGGAAGAGTGTGAATGG		
*GLUT2*	F: GCGGACTTCTGTGGACCTTATGTG	XM_005905570.2	108
	R: CCCTCTTCTTTCGGAACTCTGCTG		
*PK*	F: GCCATAATCGTCCTCACCAAGTCTG	XM_005905593.1	99
	R: CTTACACACCACAGGGAAGATGCC		
*GK*	F: TCGTTGGCTCCTTGACAATGTGAG	XM_005899746.1	107
	R: TCGTTGGCTCCTTGACAATGTGAG		
*FXR*	F: GCTGTTCTGATGGATGGGATGACTG	XM_005892526.2	102
	R: GGGAGGTTTCTTTGTCTGCTCTGAG		
*CYP7A1*	F: *AGCTGACGGAGGGCTTGAGAC*	NM_001205677.2	108
	R: AGGACTGCGAGGAGTGACTTGG		
*CYP8B1*	F: GCAGAGGAAGCTAGACTTTGTGGAG	XM_005222522.5	109
	R: GCTTGGTGCTGGCTGAGTGTATC		
*BSEP*	F: GGCACTGGACAATGAGAGCGAAG	XM_005887600.2	105
	R: GATAGGCGATGAGCGACAGAGATG		
*SHP*	F: GACGGAGGCTCAGTACAAGTTCATC	XM_005907490.2	104
	R: GTTCTTCATTGCTGGCGGGTAGG		
*LXR-α*	F: GTTTGCCTTGCTCATTGCCATCAG	XM_005898931.2	108
	R: CGGAGGCTCACCAGTTTCATCAG		
*β-actin*	F: GGTTGGATCGAGCATTCCCA R: AAAAGCGATCACCTCCCCTG	XM_005887322.2	104

Abbreviations: Toll-like receptor 4 (*TLR4*), Interleukin-8 (*IL-8*), Tumor necrosis factor (*TNF-α*), Interleukin-1β (*IL-1β*), glycogen synthase (*GS*), glucose transporter 2 (*GLUT2*), pyruvate kinase (*PK*), glucokinase (*GK*), farnesoid *X* receptor (*FXR*), cholesterol 7 alpha-hydroxylase (*CYP7A1*), sterol 12-alpha-hydroxylase (*CYP8B1*), small heterodimer partner (*SHP*), bile salt export pump (*BSEP*), liver *X* receptor α (*LXR-α*).

**Table 3:** Effects of RPG and RPT levels on growth performance in yaks.

Items	LG		HG		SEM	*p*-Values		
	LT	HT	LT	HT		G	T	G*T
Initial weight, kg	192.75	187.92	195.75	197.83	4.52	0.50	0.88	0.72
Final weight, kg	218.25	210.52	219.75	220.33	4.03	0.51	0.67	0.62
ADG, kg/d	0.40	0.36	0.38	0.36	0.02	0.77	0.42	0.79
DMI, kg/d	4.28	4.26	4.36	4.28	0.05	0.19	0.24	0.10
F/G, %	11.91	12.13	11.86	12.32	0.51	0.95	0.75	0.91

Abbreviations—LGLT: the supplemental level of RPG in the diet is 1% and the supplemental level of RPT is 5 g/d. LGHT: the added amount of RPG in the diet is 1% and the added amount of RPT is 20 g/d. HGLT: the supplemental level of RPG in the diet is 3% and the supplemental level of RPT is 5 g/d. HGHT: the addition of RPG in the diet is 3% and the addition of RPT is 20 g/d; SEM: standard error; G: RPG; T: RPT; G*T: interaction effect of RPG and RPT; ADG: average daily gain; DMI: dry matter intake; F/G: feed-to-weight ratio.

**Table 4:** Effects of RPG and RPT levels on organ index in yaks.

Items	LG		HG		SEM	*p*-Values		
	LT	HT	LT	HT		G	T	G*T
Organ weight								
Heart, kg	0.88	0.82	0.88	0.93	0.02	0.2	0.86	0.20
Liver, kg	2.47	2.31	2.40	2.52	0.05	0.46	0.87	0.15
Spleen, kg	0.46	0.43	0.43	0.41	0.01	0.47	0.44	0.82
Kidney, kg	0.40	0.36	0.39	0.39	0.01	0.56	0.22	0.14
Final weight, kg	218.25	210.52	219.75	220.33	4.03	0.51	0.67	0.62
Organ Index								
Heart, %	0.41	0.39	0.41	0.43	0.01	0.19	1.00	0.16
Liver, %	1.15	1.11	1.12	1.17	0.01	0.63	0.94	0.14
Spleen, %	0.22	0.21	0.20	0.20	0.01	0.42	0.60	0.89
Kidney, %	0.19	0.17	0.18	0.18	0.00	1.00	0.16	0.12

Abbreviations—LGLT: the supplemental level of RPG in the diet is 1% and the supplemental level of RPT is 5 g/d; LGHT: the added amount of RPG in the diet is 1% and the added amount of RPT is 20 g/d; HGLT: the supplemental level of RPG in the diet is 3% and the supplemental level of RPT is 5 g/d; HGHT: the addition of RPG in the diet is 3% and the addition of RPT is 20 g/d; SEM: standard error; G: RPG; T: RPT; G*T: the interaction effect of RPG and RPT.

**Table 5:** Effects of RPG and RPT levels on serum biochemical parameters in yaks.

Items	LG		HG		SEM	*p*-Values		
	LT	HT	LT	HT		G	T	G*T
ALP/(U/L)	68.2	69.75	62.00	69.00	4.24	0.74	0.67	0.58
AST/(U/L)	84.46	82.10	77.25	64.70	3.68	0.07	0.25	0.42
GGT/(U/L)	10.63	10.70	10.12	8.46	0.48	0.04	0.23	0.19
HDL-C/(mmol/L)	1.94	2.21	2.19	2.07	0.07	0.65	0.56	0.13
LDL-C/(mmol/L)	0.83	0.99	0.97	0.90	0.04	0.68	0.58	0.12
TC/(mmol/L)	2.47	2.95	2.97	2.67	0.12	0.61	0.67	0.08
Glu/(mmol/L)	2.86	2.66	3.29	3.17	0.09	0.00	0.27	0.79
TP/(g/L)	64.85	64.19	63.16	64.45	1.48	0.83	0.93	0.77
ALB/(g/L)	27.16	27.53	28.42	27.27	0.49	0.67	0.74	0.52
LDH/(U/L)	997.05 ^ab^	1119.25 ^a^	951.18 ^ab^	857.20 ^b^	39.83	0.01	0.59	0.03
TBA/(umol/L)	26.34	26.71	27.87	28.03	0.14	0.83	0.97	1.00

Abbreviations—LGLT: the supplemental level of RPG in the diet is 1% and the supplemental level of RPT is 5 g/d; LGHT: the added amount of RPG in the diet is 1% and the added amount of RPT is 20 g/d; HGLT: the supplemental level of RPG in the diet is 3% and the supplemental level of RPT is 5 g/d; HGHT: the addition of RPG in the diet is 3% and the addition of RPT is 20 g/d; SEM: standard error; G: RPG; T: RPT; G*T: the interaction effect of RPG and RPT; ALP: alkaline phosphatase; AST: aspartate aminotransferase; GGT: glutamyl transpeptidase; HDCL: high-density lipoprotein; LDLC: low-density lipoprotein; TC: total cholesterol; GLU: glucose; TP: total protein; ALB: albumin; LDH: lactate dehydrogenase; TBA: total bile acid. Letters a and b, when different, denote a significant difference.

**Table 6:** Effects of RPG and RPT levels on serum immunological indices in yaks.

Items	LG		HG		SEM	*p*-Values		
	LT	HT	LT	HT		G	T	G*T
IL-1β, ng/L	52.04	53.69	54.07	52.29	0.08	0.88	0.97	0.36
TNF-α, ng/L	257.72	263.52	266.26	244.26	5.44	0.54	0.51	0.58
IL-6, ng/L	71.23	16.69	16.64	17.20	0.27	0.94	0.97	0.35
IL-4, ng/L	96.55	92.33	92.78	94.82	1.49	0.84	0.73	0.33
IL-10, ng/L	42.46	41.42	45.57	44.46	0.71	0.03	0.72	0.97

Abbreviations—LGLT: the supplemental level of RPG in the diet is 1% and the supplemental level of RPT is 5 g/d; LGHT: the added amount of RPG in the diet is 1% and the added amount of RPT is 20 g/d; HGLT: the supplemental level of RPG in the diet is 3% and the supplemental level of RPT is 5 g/d; HGHT: the addition of RPG in the diet is 3% and the addition of RPT is 20 g/d; SEM: standard error; G: RPG; T: RPT; G*T: the interaction effect of RPG and RPT.

**Table 7:** Effects of RPG and RPT on the antioxidant capacity of liver.

Items	LG		HG		SEM	*p*-Values		
	LT	HT	LT	HT		G	T	G*T
AST, U/g	592.08	574.02	581.97	581.59	11.742	0.960	0.713	0.724
ALT, U/g	310.16	291.32	270.84	284.52	14.952	0.461	0.942	0.601
PCO, nmol/mgprot	5.68	3.64	4.70	2.67	0.374	0.144	0.005	0.979
SOD, U/mgprot	6.27	6.90	7.16	6.65	0.38	0.482	0.895	0.223
MDA, nmol/mgprot	2.73	2.29	3.19	2.66	0.114	0.052	0.027	0.839
CAT, U/mgprot	447.07	410.18	392.98	424.80	11.855	0.110	0.592	0.425
GSH-PX, U/mgprot	73.99	67.47	77.49	67.40	2.498	0.734	0.109	0.724
T-AOC, mmol/mL	0.85	0.89	0.70	0.87	0.03	0.168	0.091	0.271

Abbreviations—LGLT: the supplemental level of RPG in the diet is 1% and the supplemental level of RPT is 5 g/d; LGHT: the added amount of RPG in the diet is 1% and the added amount of RPT is 20 g/d; HGLT: the supplemental level of RPG in the diet is 3% and the supplemental level of RPT is 5 g/d; HGHT: the addition of RPG in the diet is 3% and the addition of RPT is 20 g/d; SEM: standard error; G: RPG; T: RPT; G*T: the interaction effect of RPG and RPT; AST: aspartate aminotransferase; ALT: alanine aminotransferase; PCO: protein carbonyl; T-SOD: total superoxide dismutase; MDA: malonaldehyde; CAT: catalase; GSH-Px: glutathione peroxidase; T-AOC: total antioxidant capacity.

## Data Availability

The original contributions presented in this study are included in the article. Further inquiries can be directed to the corresponding author(s).

## References

[1] Li Y., Zong W., Zhao S., Qie M., Yang X., Zhao Y. (2023). Nutrition and edible characteristics, origin traceability and authenticity identification of yak meat and milk: A review. Trends Food Sci. Technol..

[2] Qiu Q., Zhang G., Ma T., Qian W., Wang J., Ye Z., Cao C., Hu Q., Kim J., Larkin D.M. (2012). The yak genome and adaptation to life at high altitude. Nat. Genet..

[3] Xue B., Zhao X.Q., Zhang Y.S. (2005). Seasonal changes in weight and body composition of yak grazing on alpine-meadow grassland in the Qinghai-Tibetan plateau of China. J. Anim. Sci..

[4] Yang S., Liu J., Gu Z., Liu P., Lan Q. (2022). Physiological and metabolic adaptation to heat stress at different altitudes in yaks. Metabolites.

[5] Wang X., Zhao K., Zhao S., Zhou J., Cao M., Lu L., Chen Y., Yang H., Zhang B., Shao C. (2024). Effects of dietary rumen-protected glucose level and taurine supplementation on weight change and oxidative stress state of yaks after transport. Front. Vet. Sci..

[6] Wang Y.P., Cai M., Hua D.K., Zhang F., Jiang L.S., Zhao Y.G., Wang H., Nan X.M., Xiong B.H. (2020). Metabolomics reveals effects of rumen-protected glucose on metabolism of dairy cows in early lactation. Anim. Feed. Sci. Technol..

[7] Li X.P., Tan Z.L., Jiao J.Z., Long D.L., Zhou C.S., Yi K.L., Liu C.H., Kang J.H., Wang M., Duan F.H. (2019). Supplementation with fat-coated rumen-protected glucose during the transition period enhances milk production and influences blood biochemical parameters of liver function and inflammation in dairy cows. Anim. Feed. Sci. Technol..

[8] McCarthy C.S., Dooley B.C., Branstad E.H., Kramer A.J., Horst E.A., Mayorga E.J., Al-Qaisi M., Abeyta M.A., Perez-Hernandez G., Goetz B.M. (2020). Energetic metabolism, milk production, and inflammatory response of transition dairy cows fed rumen-protected glucose. J. Dairy. Sci..

[9] Oliveira M.W., Minotto J.B., de Oliveira M.R., Zanotto-Filho A., Behr G.A., Rocha R.F., Moreira J.C., Klamt F. (2010). Scavenging and antioxidant potential of physiological taurine concentrations against different reactive oxygen/nitrogen species. Pharmacol. Rep..

[10] Han H., Zhang J., Chen Y., Shen M., Yan E., Wei C., Yu C., Zhang L., Wang T. (2020). Dietary taurine supplementation attenuates lipopolysaccharide-induced inflammatory responses and oxidative stress of broiler chickens at an early age. J. Anim. Sci..

[11] Faghfouri A.H., Shoura S.M.S., Fathollahi P., Shadbad M.A., Papi S., Ostadrahimi A., Faghfuri E. (2022). Profiling inflammatory and oxidative stress biomarkers following taurine supplementation: A systematic review and dose-response meta-analysis of controlled trials. Eur. J. Clin. Nutr..

[12] Inam-u-Ilah, Piao F.Y., Aadil R.M., Suleman R., Li K.X., Zhang M.R., Wu P.A., Shahbaz M., Ahmed Z. (2018). Ameliorative effects of taurine against diabetes: A review. Amino Acids..

[13] Shi Y., Zhong L., Zhong H., Zhang J., Che C., Fu G., Hu Y., Mai K. (2022). Taurine supplements in high-fat diets improve survival of juvenile Monopterus albus by reducing lipid deposition and intestinal damage. Aquaculture.

[14] NRC (2007). Nutrient Requirements of Small Ruminants: Sheep, Goats, Cervids and New World Camelids.

[15] Tilley J.M.A., Terry R.A. (1963). A two-stage technique for the in vitro digestion of forage crops. Grass Forage Sci..

[16] Zhang S., Hu J., Liu Y., Shen X., Liu C., Cheng L., Li M., Zhao G. (2024). Taurine drives body protein renewal and accretion in beef steers. Anim. Nutr..

[17] Kumkhong S., Marandel L., Plagnes-Juan E., Veron V., Boonanuntanasarn S., Panserat S. (2020). Glucose Injection into Yolk Positively Modulates Intermediary Metabolism and Growth Performance in Juvenile Nile Tilapia (*Oreochromis niloticus*). Front. Physiol..

[18] Chen J., Zhang X., Zhang Y., Jiang S., Han Y., Zhang L., Zhang Y., Du H. (2024). Taurine enhances growth performance by improving intestinal integrity and antioxidant capacity of weaned piglets. J. Anim. Sci..

[19] Li X., Wu J., Zhuang Z., Ye Y., Zhou S., Qiu Y., Ruan D., Wang S., Yang J., Wu Z. (2023). Integrated single-trait and multi-trait GWASs reveal the genetic architecture of internal organ weight in pigs. Animals.

[20] Makris K., Mousa C., Cavalier E. (2023). Alkaline Phosphatases: Biochemistry, Functions, and Measurement. Calcif. Tissue Int..

[21] Ozer J., Ratner M., Shaw M., Bailey W., Schomaker S. (2008). The current state of serum biomarkers of hepatotoxicity. Toxicology.

[22] De Grandi A., Franzini M., Rosipal S., Rosipal R., Debreova M., Corti A., Ruetzler-Dichtl E., Scholl-Buergi S., Paolicchi A., Pompella A. (2020). Highly elevated plasma γ-Glutamyltransferase elevations: A trait caused by γ-Glutamyltransferase 1 transmembrane mutations. Hepatology.

[23] Hagar H.H. (2004). The protective effect of taurine against cyclosporine A-induced oxidative stress and hepatotoxicity in rats. Toxicol. Lett..

[24] Lin W.C., Hoe B.C., Li X., Lian D., Zeng X. (2024). Glucose metabolism-modifying natural materials for potential feed additive development. Pharmaceutics.

[25] Wang Y., Zhao Y., Nan X., Wang Y., Cai M., Jiang L., Luo Q., Xiong B. (2022). Rumen-protected glucose supplementation alters fecal microbiota and its metabolic profiles in early lactation dairy cows. Front. Microbiol..

[26] Li S., Tan H.Y., Wang N., Zhang Z.J., Lao L., Wong C.W., Feng Y. (2015). The Role of Oxidative Stress and Antioxidants in Liver Diseases. Int. J. Mol. Sci..

[27] Yu Z., Wu X., Zheng L., Dai Z., Wu L. (2020). Effect of acute exposure to ammonia and BFT alterations on *Rhynchocypris lagowski*: Digestive enzyme, inflammation response, oxidative stress and immunological parameters. Environ. Toxicol. Pharmacol..

[28] Akagawa M. (2021). Protein carbonylation: Molecular mechanisms, biological implications, and analytical approaches. Free Radic. Res..

[29] Rosemberg D.B., Da R.R., Rico E.P., Zanotto-Filho A., Dias R.D., Bogo M.R., Bonan C.D., Moreira J.C., Klamt F., Souza D.O. (2010). Taurine prevents enhancement of acetylcholinesterase activity induced by acute ethanol exposure and decreases the level of markers of oxidative stress in zebrafish brain. Neuroscience.

[30] Zhang Y., Wei Z., Yang M., Liu D., Pan M., Wu C., Zhang W., Mai K. (2021). Dietary taurine modulates hepatic oxidative status, ER stress and inflammation in juvenile turbot (*Scophthalmus maximus* L.) fed high carbohydrate diets. Fish. Shellfish. Immunol..

[31] Moroz L.A., Talako T.M., Potapnev M.P., Soroka N.F. (2019). Dichotomy of local Th1-and systemic Th2/Th3-dependent types of Immune response in rheumatoid arthritis. Bull. Exp. Biol. Med..

[32] Cantuaria A., Figueiredo T.M., Freire M.S., Lima S., Almeida J.A., Franco O.L., Rezende T. (2018). The effects of glucose concentrations associated with lipopolysaccharide and interferon-gamma stimulus on mediators’ production of RAW 264.7 cells. Cytokine.

[33] Kawai T., Akira S. (2011). Toll-like Receptors and Their Crosstalk with Other Innate Receptors in Infection and Immunity. Immunity.

[34] Arnold H., Pluta H.J., Braunbeck T. (1996). Sublethal effects of prolonged exposure to disulfoton in rainbow trout (*Oncorhynchus mykiss*): Cytological alterations in the liver by a potent acetylcholine esterase inhibitor. Ecotoxicol. Environ. Saf..

[35] Ghosh S., Chowdhury S., Das A.K., Sil P.C. (2019). Taurine ameliorates oxidative stress induced inflammation and ER stress mediated testicular damage in STZ-induced diabetic Wistar rats. Food Chem. Toxicol..

[36] Lin C., Chiu C., Chen Y., Chen M., Hsu T., Tzang B. (2015). Taurine Attenuates Hepatic Inflammation in Chronic Alcohol-Fed Rats Through Inhibition of TLR4/MyD88 Signaling. J. Med. Food..

[37] Ma Y., Zhang Y., Li R., Deng S., Qin Q., Ran C., Hao Y., Zhang J., Zhu L. (2022). Mechanism of taurine reducing inflammation and organ injury in sepsis mice. Cell Immunol..

[38] Hu D., Wang H., Huang X., Jiang Y., Qin Y., Xiong B., Qin G., Sooranna S.R., Pinhu L. (2016). Investigation of association between IL-8 serum levels and IL8 polymorphisms in Chinese patients with sepsis. Gene.

[39] Wang S., Fang H., Xie J., Wu Y., Tang Z., Liu Z., Lv J., Yu J. (2021). Physiological responses of cucumber seedlings to different supplemental light duration of red and blue LED. Front. Plant Sci..

[40] Kierans S.J., Taylor C.T. (2024). Glycolysis: A multifaceted metabolic pathway and signaling hub. J. Biol. Chem..

[41] Wang K., Shi M., Luk A.O.Y., Kong A.P.S., Ma R.C.W., Li C., Chen L., Chow E., Chan J.C.N. (2024). Impaired GK—GKRP interaction rather than direct GK activation worsens lipid profiles and contributes to long-term complications: A Mendelian randomization study. Cardiovasc. Diabetol..

[42] von Wilamowitz-Moellendorff A., Hunter R.W., Garcia-Rocha M., Kang L., Lopez-Soldado I., Lantier L., Patel K., Peggie M.W., Martinez-Pons C., Voss M. (2013). Glucose-6-Phosphate-Mediated activation of liver glycogen synthase plays a key role in hepatic glycogen synthesis. Diabetes.

[43] Noguchi T., Inoue H., Tanaka T. (1986). The M1- and M2-type isozymes of rat pyruvate kinase are produced from the same gene by alternative RNA splicing. J. Biol. Chem..

[44] Zhang Y., Xie S., Guo T., Liu Z., Fang H., Zheng L., Xie J., Tian L., Liu Y., Jin N. (2020). High dietary starch inclusion impairs growth and antioxidant status, and alters liver organization and intestinal microbiota in largemouth bass Micropterus salmoides. Aquac. Nutr..

[45] Qin G., Pan M., Huang D., Li X., Liu Y., Yu X., Mai K., Zhang W. (2024). The Molecular Mechanism of Farnesoid X Receptor Alleviating Glucose Intolerance in Turbot (*Scophthalmus maximus*). Cells.

[46] Buschiazzo H., Exton J.H., Park C.R. (1970). Effects of glucose on glycogen synthetase, phosphorylase, and glycogen deposition in the perfused rat liver. Proc. Natl. Acad. Sci. USA.

[47] Perino A., Demagny H., Velazquez-Villegas L., Schoonjans K. (2021). Molecular physiology of bile acid signaling in health, disease, and aging. Physiol. Rev..

[48] Boyer J.L. (2013). Bile formation and secretion. Compr. Physiol..

[49] Berrabah W., Aumercier P., Gheeraert C., Dehondt H., Bouchaert E., Alexandre J., Ploton M., Mazuy C., Caron S., Tailleux A. (2014). Glucose sensing O-GlcNAcylation pathway regulates the nuclear bile acid receptor farnesoid X receptor (FXR). Hepatology.

