# Correction: Chen et al. Effects of Dietary Rumen-Protected Glucose and Rumen-Protected Taurine Levels on Growth Performance, Serum Biochemical Indicators, and Liver Health in Yaks. *Animals* 2025, *15*, 1152

**DOI:** 10.3390/ani15213212

**Published:** 2025-11-05

**Authors:** Yuanyuan Chen, Xiaolin Wang, Lianghao Lu, Bao Zhang, Huaming Yang, Shoupei Zhao, Zhisheng Wang, Lizhi Wang, Quanhui Peng, Bai Xue

**Affiliations:** Animal Nutrition Institute, Sichuan Agricultural University, Chengdu 611130, China; cyy2022214041@stu.sicau.edu.cn (Y.C.); wxl547312630@163.com (X.W.); 13551012639@163.com (L.L.); 2022314074@stu.sicau.edu.cn (B.Z.); 13980277829@163.com (H.Y.); 2023114011@stu.sicau.edu.cn (S.Z.); zswangsicau@126.com (Z.W.); 1285@sicau.edu.cn (L.W.); pengquanhui@126.com (Q.P.)

**Text Correction**

There was an error in the original publication [1].

1. Upon verification, we found a typographical error in the data regarding the weight of the experimental subjects in the abstract. The weight should be corrected from “170 ± 10.4 kg” to “192.7 ± 20.6 kg.”

Yaks are an important livestock species on the Tibetan Plateau, but traditional grazing practices cause a sharp drop in their weight during winter, leading to grassland degradation due to overgrazing. Although off-site fattening can improve performance and protect ecology, it often leads to a negative energy balance, liver metabolism disorders, and immune impairment due to stress. However, the effects of rumen-protected glucose (RPG) and rumen-protected taurine (RPT) on yak liver health are not yet clear. The purpose of this study was to evaluate the effects of dietary RPG and RPT levels on the growth performance, serum biochemical parameters, liver antioxidant capacity, and immunity of yaks. Twenty-eight healthy yaks weighing 192.7 ± 20.6 kg were randomly divided into four treatments: LGLT (RPG: 1%—low RPG [LG]; RPT: 5 g/d—low RPT [LT]), LGHT (RPG: 1%—low RPG [LG]; RPT: 20 g/d—high RPT [HT]), HGLT (RPG: 3%—high RPG [HG]; RPT: 5 g/d—low RPT [LT]), and HGHT (RPG: 3%—high RPG [HG]; RPT: 20 g/d—high RPT [HT]). The results showed that compared with the LTHT treatment group, the HGHT group upregulated the serum concentrations of glucose (*p* = 0.004) and Interleukin-10 (*p* = 0.03), the relative mRNA expression of small heterodimer partners (*p* = 0.01), and the sterol 12-alpha-hydroxylase (*p* < 0.001), while reducing the serum concentration of gamma-glutamyl transferase (*p* = 0.048). The serum concentration of hepatic protein carbonyl (*p* = 0.005) and malondialdehyde (*p* = 0.03) was lower in the LGHT and HGHT treatment groups than in the LGLT and HGLT groups. The relative mRNA expression of Toll-like receptor 4 (*p* = 0.02), Interleukin-8 (*p* < 0.01), and Interleukin-1β (*p* < 0.01) was lower in the LGHT and HGHT groups than in the LGLT and HGLT groups. Tumor necrosis factor expression was lower (*p* = 0.04) and glucose transporter 2 expression was higher (*p* < 0.01) in the HGHT group compared to other treatment groups. The expression of glucokinase, glycogen synthase, pyruvate kinase, and farnesoid X receptor was higher in the HGLT treatment group than in other treatments (*p* < 0.01). In conclusion, dietary supplementation with 3% PRG and 5 g/d PRT can enhance liver antioxidant capacity and immune function, reduce lipid peroxidation, and promote glucose and bile acid metabolism in yaks.

2. In Section 2.2 Determination of Growth Performance: The term “average feed intake (ADIF)” should be corrected to “dry matter intake (DMI)”.

Yaks were weighed and recorded on days 0 and 63 of the experiment. This ensured that the amount of leftover feed on the same day was 10%; then, the feed intake of the test yaks in each group was recorded, and the average daily gain (ADG), dry matter intake (DMI), and feed-to-weight ratio (F/G) were calculated.

3. In Section 3.1 Growth Performance: The abbreviation “ADFI” should be corrected to “DMI”.

The level of RPG and RPT in the diet did not affect the final BW, ADG, DMI, and F/G in yaks (*p* > 0.05; Table 3). In addition, there was no significant interaction between RPG and RPT regarding the final weight, ADG, DMI, and F/G (*p* > 0.05).

4. In Section 4 Discussion: The abbreviation “ADFI” should be corrected to “DMI”.

In the process of animal production, growth performance is an important index. Zhang et al. [16] demonstrated that supplementing the diet with RPT can enhance the ADG of steers. Early glucose injection increased the body weight of *Oreochromis niloticus* at 12–16 weeks, thus improving the growth performance of the fish [17]. Dietary supplementation with 0.3% or 0.5% taurine improves the growth performance of weaned piglets by increasing the daily weight gain and the feed-to-gain ratio [18]. However, Wang et al. [5] found that supplementing yaks with RPG and RPT after long-distance transportation did not result in any changes in growth performance. In our study, increasing the concentrations of RPG and RPT in diets did not change the ADG, DMI, and F/G of yaks, suggesting that the dosage of RPG and RPT is not the main reason for affecting growth performance. We did not provide data on non-RPG and -RPT diets, so we cannot prove whether feeding these two additives improves yak growth performance compared with not feeding, which will be refined in future work.

The authors state that the scientific conclusions are unaffected. This correction was approved by the Academic Editor. The original publication has also been updated.
